# Supplementary material for: Okadaic Acid Activates JAK/STAT Signaling to Affect Xenobiotic Metabolism in HepaRG Cells
Source: Cells. 2023 Feb 28;12(5):770. doi: 10.3390/cells12050770 (PMC10000888; doi:10.3390/cells12050770)
Supplement: Supplementary file 1 [file cells-12-00770-s001.zip › cells-2234378-supplementary.pdf]

Table S1. Used primer sequences for the qPCR.

Protein name	Forward primer	Reverse primer
IL-1α	TCTTCTGGGAAACTCACGGC	GCACACCCAGTAGTCTTGCT
IL-1β	GGCTGCTCTGGGATTCTCTTC	ACTGGCGAGCTCAGGTACT
IL-6	AGTGAGGAACAAGCCAGAGC	GCATTTGTGGTTGGGTCAGG
IL-8	GACATACTCCAAACCTTTCCACC	AATTTCTGTGTTGGCGCAGTG
IL-12	CCTGGACCACCTCAGTTTGG	AGGCATGGGAACATTCCTGG
TNFα	CTGGGCAGGTCTACTTTGGG	GAGCCAGAAGAGGTTGAGGG
SOCS3	CTGCCTCAATCACTCTGTCTTTT	TCAAGCATCTCCTAATAGCCTCA
CYP1A1	ACCCTGAAGGTGACAGTTCC	TCTTGGAGGTGGCTGAGGTA
CYP2B6	TTCGGCGATTCTCTGTGACC	ATGAGGGCCCCCTTGGAT
CYP3A4	TCACAAACCGGAGGCCTTTT	TGGTGAAGGTTGGAGACAGC
GAPDH	TTAAAAGCAGCCCTGGTGAC	CTCTGCTCCTCCTGTTCGAC
GUSB	CACCAGGGACCATCCAATAC	ATGTAGGTGGTGGGTGTCGT
PXR	GGCATGAAGAAGGAGATGAT	TGGGAGAAGGTAGTGTCAAA
RXRα	GGGCTGGGACTGTTTCGTTT	CATCGTCTGTCCTGGCGTTT

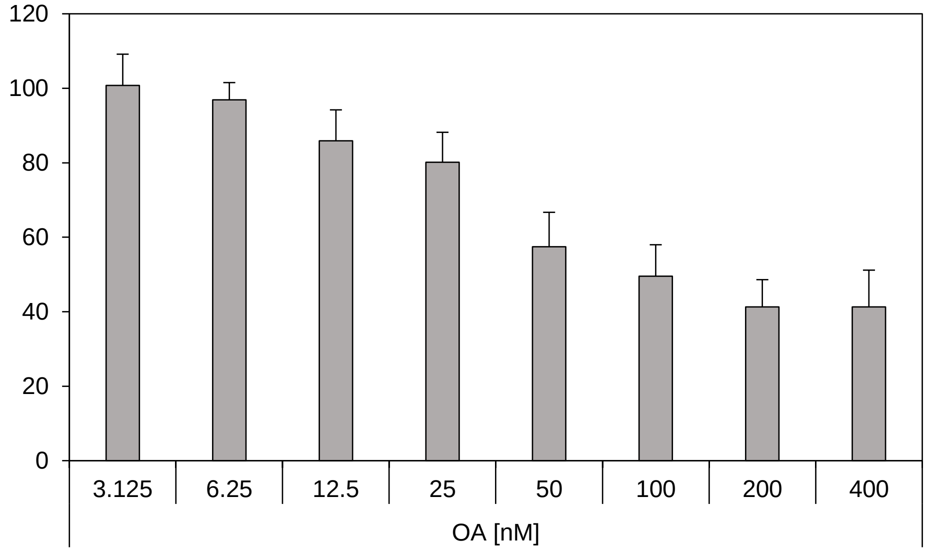

Figure S1. CellTiter-Blue® Cell Viability Assay of OA in HEK-T cells. Cell viability was determined in HEK-T cells after 24 h treatment with OA using the CellTiter-Blue® Cell Viability Assay (Promega, Madison, WI, USA). CellTiter-Blue® reagent was diluted 1:4 with PBS, and 20 µl of the diluted reagent were added to each well. The cells were incubated for 2 h at 37°C, and the fluorescence was measured at 590 nm (excitation at 540 nm) using an Infinite M200 microplate reader (Tecan). OA concentrations between 3.125 and 400 nM were tested. Concentrations between 1.56 and 50 nM were chosen as non-toxic concentrations for analysis.

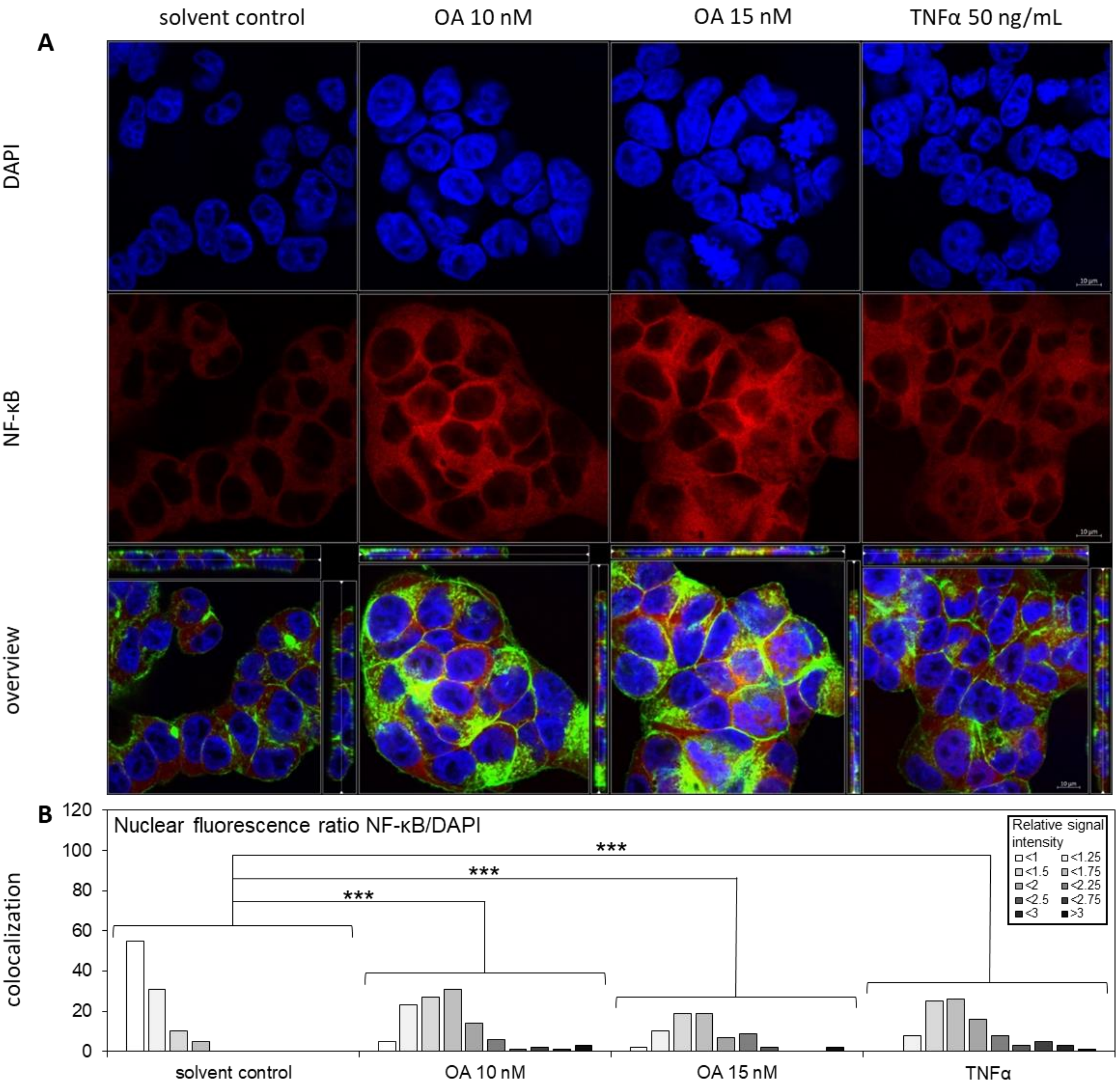

Figure S2. Activation of NF-κB in HepG2 cells. HepG2 cells were used for method development. HepG2 cells were incubated with 10 nM OA, 15 nM OA, 50 ng/ml TNFα or the respective solvent control for 24h. **A**: Nuclei were stained with DAPI and the actin cytoskeleton was stained using ActinGreen™ 488 ReadyProbes™ reagent. Immunostaining of NF-κB was carried out using a primary antibody against NF-κB and stained using a secondary antibody conjugated with the fluorophore Alexa Fluor™ 633. Fluorescence was detected using a confocal laser scanning microscope at ex wavelengths of 405 nm (DAPI, blue), 488 nm (ActinGreen, green), and 633 nm (NF-κB, red). Z-stacks spanning through the entire cell layer were recorded at 63x magnification. Brightness was increased for the blue and red channels for 40% in each image. **B**: The nuclear fluorescence ratio was determined using ImageJ 1.53e. Nuclei were marked as ROI and the fluorescence intensity of the red and blue channels in each ROI was determined separately. A ratio of NF-κB/DAPI signal intensity was then formed and normalized to the mean of the solvent controls. The nuclei were divided into groups based on their signal intensity. Results show the number of nuclei per signal intensity group for each treatment group. Between 70 and 113 nuclei were evaluated per condition. Statistical analysis was performed using Wilcoxson rank-sum test (**p* < 0.05; ***p* < 0.01; ****p* < 0.001).

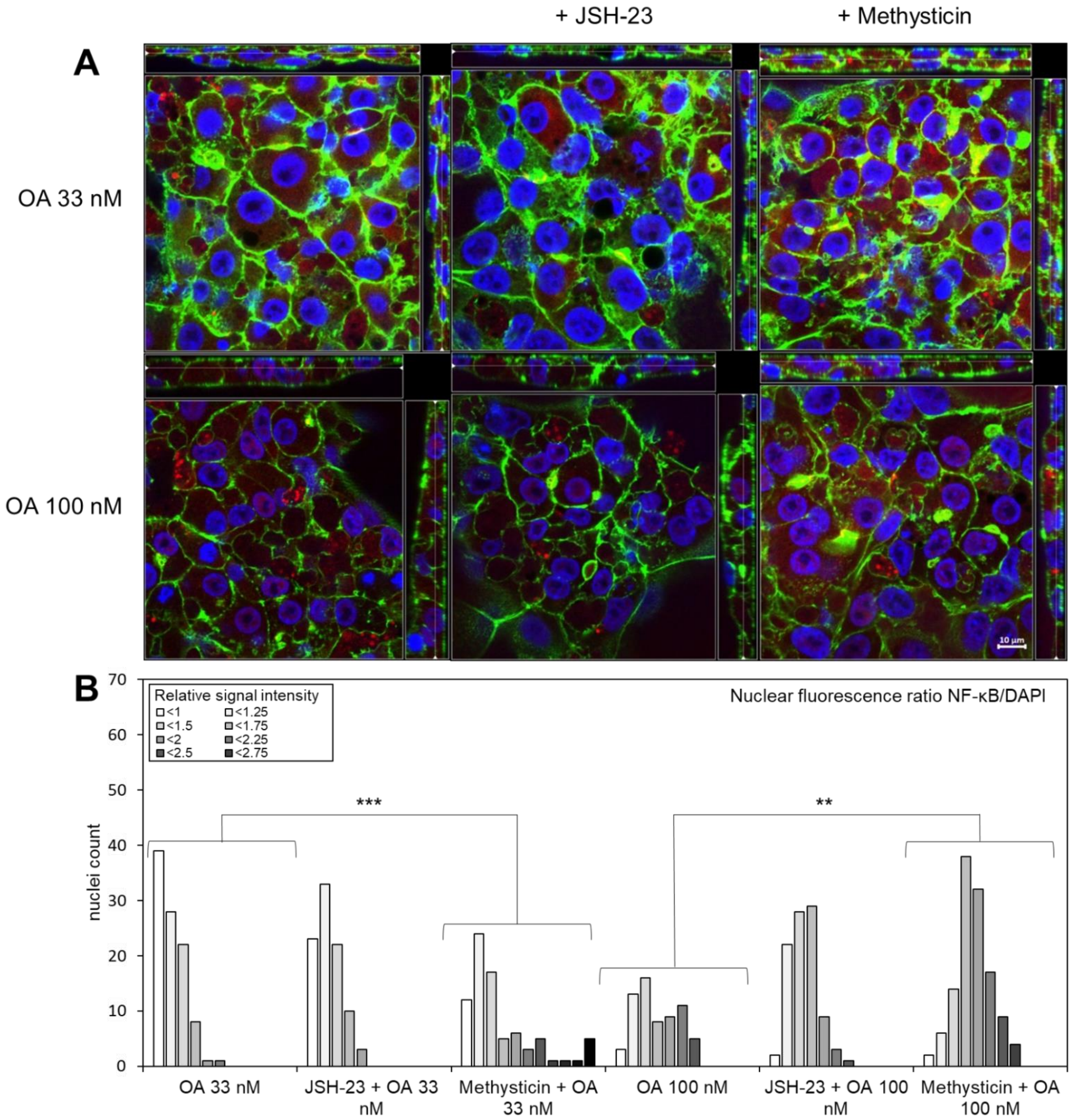

Figure S3. Activation of NF-κB in HepaRG cells after incubation with 33 and 100 nM OA in combination with NF-κB-inhibitors JSH-23 and Methysticin. **A:** Nuclei were stained with DAPI and the actin cytoskeleton was stained using ActinGreen™ 488 ReadyProbes™ reagent. Immunostaining of NF-κB was carried out using a primary antibody against phosphorylated STAT3 and stained using a secondary antibody conjugated with the fluorophore Alexa Fluor™ 633. Fluorescence was detected using a confocal laser scanning microscope at ex wavelengths of 405 nm (DAPI, blue), 488 nm (ActinGreen, green), and 633 nm (NF-κB, red). Z-stacks spanning through the entire cell layer were recorded at 63x magnification. Brightness was increased for the green channel for 15%, the blue channel for 60% and for the red channel for 70% in each image. **B:** The nuclear fluorescence ratio was determined using ImageJ 1.53e. Nuclei were marked as ROI and the fluorescence intensity of the red and blue channels in each ROI was determined separately. A ratio of NF-κB/DAPI signal intensity was then formed and normalized to the mean of the solvent controls. The nuclei were divided into groups based on their signal intensity. Results show the number of nuclei per signal intensity group for each treatment group. Between 65 and 123 nuclei were evaluated per condition. Statistical analysis was performed using Wilcoxson rank-sum test (*$p < 0.05$; **$p < 0.01$; ***$p < 0.001$).

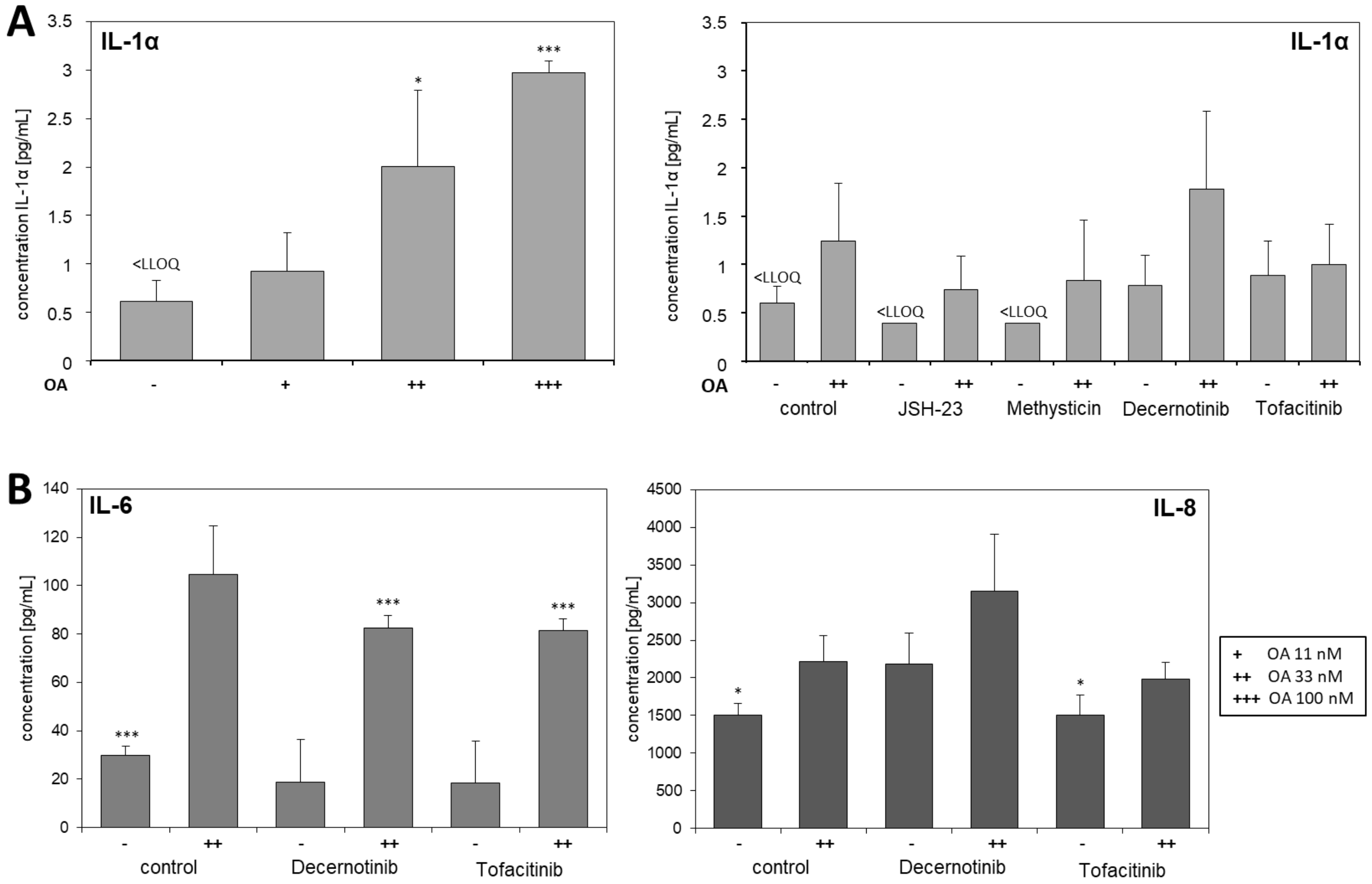

Figure S4. Release of Il-1α, IL-6 and IL-8 from HepaRG cells into the cell culture supernatant after exposure to OA. The IL-content in the cell culture supernatant was quantified using Luminex multiplex assay. **A:** Release of IL-1α into the cell culture supernatant after exposure to OA alone or in combination with the NF-κB-inhibitors JSH-23 or Methysticin and with the JAK-inhibitors Decernotinib and Tofacitinib. **B:** Release of IL-6 and IL-8 after incubation with OA in combination with the JAK-inhibitors. Statistical analysis (n=3) was performed against the 33 nM OA sample using one-way ANOVA followed by Dunnett's post-hoc test (*$p < 0.05$; **$p < 0.01$; ***$p < 0.001$). LLOQ IL-1α_ 0,79 pg/ mL IL-6: 12.89 pg/mL; LLOQ IL-8: 2.54 pg/mL.

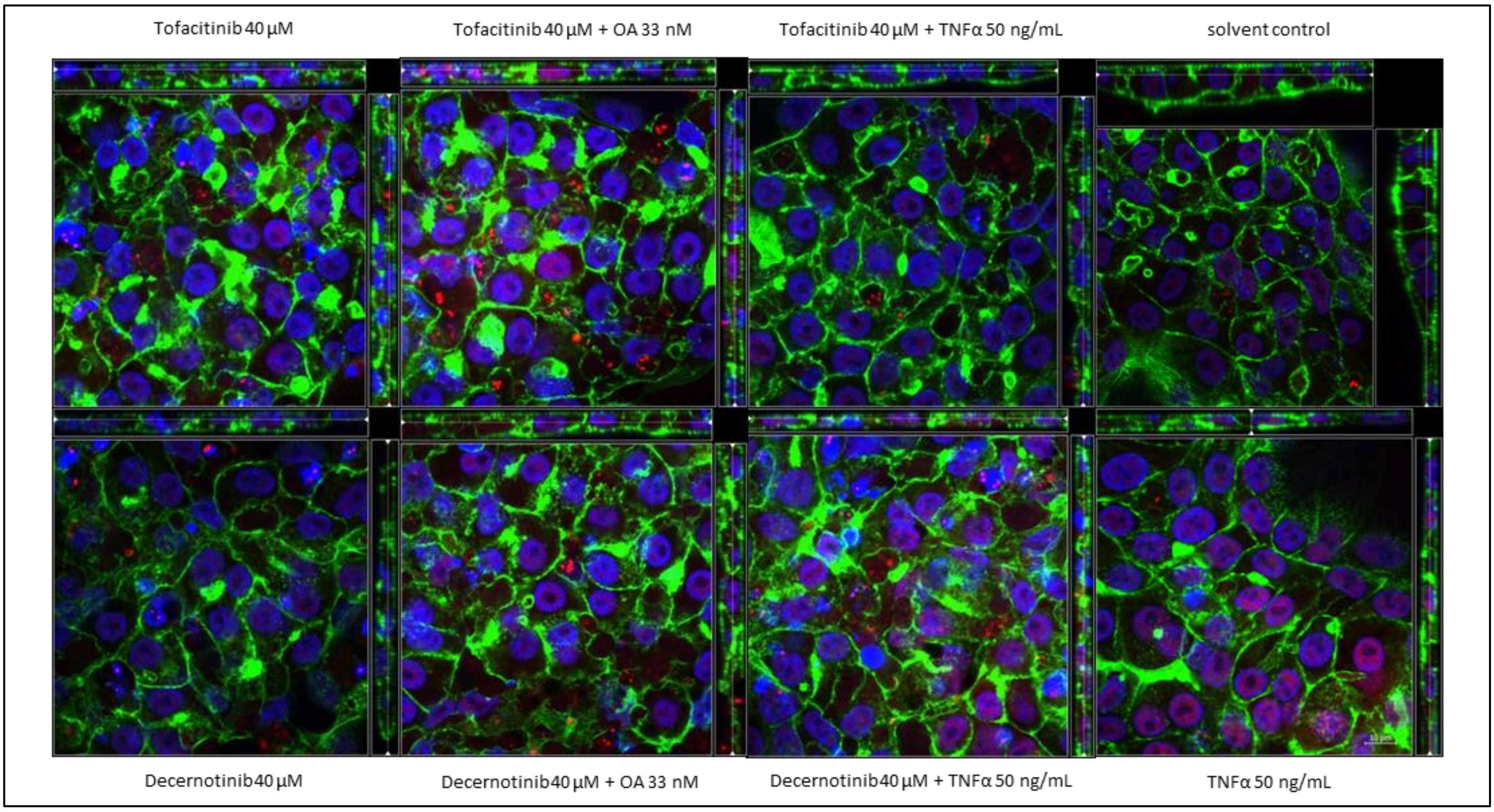

Figure S5. Activation of STAT3 in HepaRG cells. Differentiated HepaRG cells were incubated with 33 nM OA, 50 ng/ml TNFα, 40 µM Decernotinib and 40 µM Tofacitinib alone or in combination or the respective solvent control for 24h. Nuclei were stained with DAPI and the actin cytoskeleton was stained using ActinGreen™ 488 ReadyProbes™ reagent. Immunostaining of phosphoSTAT3 was carried out using a primary antibody against phosphoSTAT3 and stained using a secondary antibody conjugated with the fluorophore Alexa Fluor™ 633. Fluorescence was detected using a confocal laser scanning microscope at ex wavelengths of 405 nm (DAPI, blue), 488 nm (ActinGreen, green), and 633 nm (phosphoSTAT3, red). Z-stacks spanning through the entire cell layer were recorded at 63x magnification. Brightness was increased for the blue and red channels for 40% in each image.

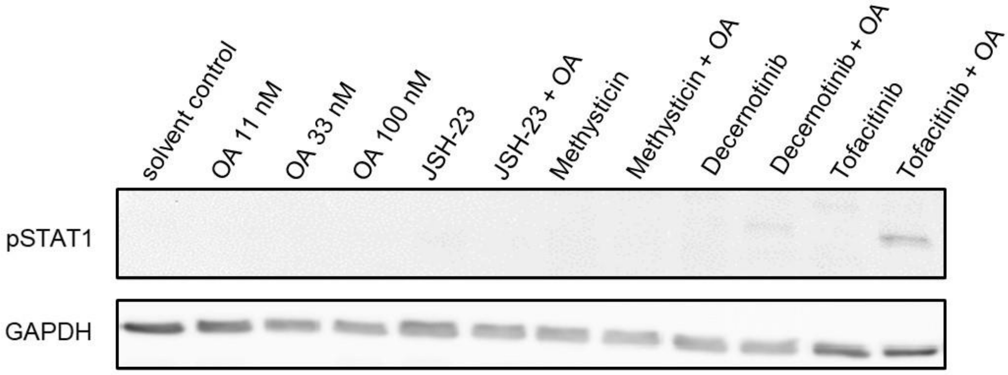

Figure S6. Western Blot of lysed HepaRG cells against phosphorylated STAT1. Differentiated HepaRG cells were treated with 33 and 100 nM OA, alone or 33 µM OA in combination with 30 µM JSH-23, or 30 µM Methysticin, two NF-κB inhibitors, or with 40 µM Decernotinib, or 40 µM Tofacitinib, two JAK activation inhibitors or with the respective solvent control for 24h. Western Blot were obtained using the wet blot method. 3 biological replicates were independently analyzed and normalized against the housekeeper GAPDH.
